# Hypoglycemia does not affect the progression of preclinical atherosclerosis in subjects with type 2 diabetes

**DOI:** 10.1371/journal.pone.0212871

**Published:** 2019-03-05

**Authors:** Concetta Irace, Antonio Cutruzzolà, Delia Francesca Carbotti, Simona Mastroianni, Michela Cavallo, Agostino Gnasso

**Affiliations:** 1 Department of Health Science, University Magna Graecia, Catanzaro, Italy; 2 University Magna Graecia, School of Medicine, Catanzaro, Italy; 3 Department of Clinical and Experimental Medicine, University Magna Graecia, Catanzaro, Italy; Hospital de la Santa Creu i Sant Pau (Universitat Autonoma de Barcelona, CIBERBBN), SPAIN

## Abstract

**Introduction:**

Intensive treatment aimed at achieving optimal metabolic control to prevent the development of chronic diabetic complications is often associated with an increased rate of hypoglycemic events. Hypoglycemia is believed to be responsible for acute fatal and nonfatal cardiovascular events likely as a consequence of the activation of pro-inflammatory and pro-atherothrombotic pathways. Hypoglycemia has been reported to influence the development of preclinical atherosclerosis. The present study was designed to prospectively evaluate whether hypoglycemia influences the function and the morphology of the arteries in subjects with type 2 diabetes without complications and uncontrolled diabetes.

**Material and methods:**

Seventy-six subjects underwent a noninvasive evaluation of carotid wall thickness and brachial artery function at baseline and after one year of treatment with the intent of obtaining optimal glycemic control. At the end of the observation time, subjects were divided in two groups: with hypoglycemia (*H-group*) or without hypoglycemia (*C-group*).

**Results:**

Baseline characteristic were comparable between groups. HbA1c significantly decreased in both groups, and fasting plasma glucose was only significant in the *H-group*. Subjects with hypoglycemia showed a significant reduction of carotid wall thickness after one-year of treatment (*H-groups*: right baseline 834±141 vs. 1-year 770±132 μ p<0.05; *C-group*: 757±162 vs. 767±135 μ p = ns). Endothelial function remained unchanged during the study for both groups.

**Discussion:**

The present findings demonstrate that hypoglycemia does not affect endothelial function. Furthermore, subjects who experience more hypoglycemia show significant reduction of carotid wall thickness. Optimal metabolic control should be pursued as soon as possible.

## Introduction

Diabetes is a progressive disease with a significant risk for long-term complications, which can be delayed or prevented by early and stable glycemic control. Large clinical trials have shown that microvascular complications are responsive to intensive treatment in both type 1 and type 2 diabetes. The progression of macrovascular complications seems to be influenced by intensive treatment in type 1 diabetes at any stage of the disease (DCCT/EDIC) and in type 2 when an early and adequate blood glucose control is achieved (UKPDS follow-up) [1,2]. The intensification of treatment is often associated with an increased incidence of hypoglycemia as described in three large clinical studies ACCORD, VADT, and ADVANCE published in the last decade [3–5]. These trials examined the effect of intensive glycemic treatment on cardiovascular morbidity and mortality in subjects with type 2 diabetes. In particular, ACCORD reported an increased risk of mortality among subjects randomized into the intensive compared with the conventional arm. The greater number of hypoglycemic events recorded in intensive treatment subjects has been hypothesized to be responsible for the increased rate of acute vascular events. However, a retrospective analysis of the ACCORD study reported increased mortality in subjects experiencing symptomatic severe hypoglycemia independent of the assignment in the intensive or standard arm treatment. Furthermore, subjects in the intensive arm with severe hypoglycemia requiring medical assistance had a lower risk of death compared with subjects in the standard arm. Even if limited by the design of the study, this retrospective analysis reveals the complex relationship between mortality and hypoglycemia [6,7]. Hypoglycemia causes pathophysiological effects on the cardiovascular system as increases in heart rate, peripheral blood pressure, and myocardial contractility and decreases in central blood pressure. In addition, hypoglycemia also has harmful effects on cardiac electrophysiology revealed as flattening or inversion of T wave, QT prolongation, and ST depression [8]. Hypoglycemia is the other unavoidable side of the coin of glucose-lowering therapy, mainly in insulin-treated patients. The rate of hypoglycemia in real life ranges from 35 to 70 per 100 patients per year, and the major determinants are sex, age, disease duration, and type of treatment [9]. In type 1 diabetes, the rate is definitely higher and strictly dependent on the intensity of treatment [10]. The fear of hypoglycemia gives rise to inadequate metabolic control. Indeed, both patients and physicians keep fasting plasma glucose at higher levels to minimize the risk of hypoglycemia. Hypoglycemia modulates the levels of cytokines, coagulation molecules and fibrinolysis factors, promoting the development of early atherosclerotic lesions [11]. Furthermore, hypoglycemia seems to influence the morphology and function of the artery. Two papers published to date have reported reduced brachial artery dilation and increased thickness of the carotid artery wall in adult subjects with type 1 and type 2 diabetes and frequent hypoglycemia [12,13].

Based on this finding, the present study was planned to evaluate whether hypoglycemia is associated with a 1-year change in carotid artery wall thickness, and percent change in brachial artery dilation was evaluated using the FMD (flow mediated dilation) technique in a sample of patients with type 2 diabetes treated to obtain the recommended HbA1c in absence of overt complications and longstanding uncontrolled hyperglycemia at the time of recruitment.

## Materials and methods

This is a cohort prospective study including adult type 2 diabetes subjects regularly attending an outpatient clinic. Exclusion criteria were previous cardiovascular events or revascularization, carotid artery stenosis >50%, peripheral artery disease, history of micro- and macroalbuminuria or CKD (chronic kidney disease), retinopathy, neuropathy, and history of severe hypoglycemia defined as an event characterized by profound neuroglycopenia requiring the assistance of another person for recovery. Longstanding uncontrolled hyperglycemia defined as three consecutive HbA1c measurements >8.5% was also considered exclusion criteria. Study subjects were recruited in a six-month period. Before enrollment, subjects were instructed about the aim of the research, and those who gave their signed informed consent and met inclusion and exclusion criteria were enrolled. Per routine practice, subjects attending the outpatient clinic undergo annual screening for micro- and macrovascular complications. Therefore, the presence of exclusion criteria was evaluated by examining clinical records.

During the first visit (*Baseline Visit*), subjects underwent clinical examination, body weight and height measurement, and blood pressure measurement. Body mass index (BMI) was calculated as weight (kg)/height (m^2^). Medical history and ongoing pharmacological treatments were also recorded. Furthermore, a blood sample was withdrawn, and vascular study was performed. The *Follow-Up visit* was scheduled 12 months later during which all subjects underwent the same procedure performed during *Baseline Visit*. One (6-month since *Baseline Visit*) additional visit was planned to evaluate self-monitoring blood glucose (SMBG) measurement to record the number of hypoglycemic events and if necessary, modify ongoing treatment to obtain desirable HbA1c value. At the *Baseline Visit*, all patients were instructed to monitor their blood glucose level at least three days per month and five times (fasting, before and 2 h after lunch and dinner) per day. Patients were also instructed to monitor blood glucose at any time of the day if typical signs or symptoms of hypoglycemia occurred.

Any symptomatic event accompanied by a measure of glucose concentration ≤ 70 mg/dL (≤ 3.9 mmol/L) was defined as ‘documented symptomatic hypoglycemia’; any blood glucose measurement ≤ 70 mg/dL (≤ 3.9 mmol/L) not accompanied by typical symptoms of hypoglycemia was defined as ‘asymptomatic hypoglycemia’.

The research was conducted in accordance with the Declaration of Helsinki and approved by the local Ethical Committee ‘Calabria Area Centro’.

Blood lipids (total cholesterol, HDL-cholesterol, triglycerides), fasting glucose and glycated hemoglobin (HbA1c) were measured at *Baseline* and *Follow-up Visits*. Lipids were measured with a commercially available kit, and fasting plasma glucose (FPG) was measured by the glucose-hexokinase method (Roche, Basel, Switzerland). HbA1c was measured by high-performance liquid chromatography standardized and aligned to the DCCT/UKPDS (Menarini, Florence, Italy).

### Carotid artery wall thickness measurement (IMT)

Common carotid artery wall thickness measurement or intima plus media thickness measurement (IMT) was performed at *Baseline* and *Follow-up Visits*. The scans were performed by an expert sonographer blinded to the clinical characteristics of the patients. An echo Doppler Philips HD 11 XE (Royal Philips Electronics, Netherlands) equipped with a 12–3 MHz linear array, and simultaneous ECG recording was used to perform the vascular study. A preliminary scan was performed to evaluate the presence of arterial plaque. Plaque was defined as a focal lesion encroaching in the lumen of at least 0.5 mm or 50% of the surrounding IMT value or as focal thickening ≥1.5 mm measured from the media-adventitia interface to the intima-lumen interface [14]. Internal carotid artery stenosis was defined as the presence of spectral broadening and normal systolic peak velocity (stenosis <50%) or presence of spectral broadening and systolic peak velocity ≥140 cm/s (stenosis >50%); occlusion was defined as the absence of Doppler signal [15]. The common carotid artery of each side was evaluated in three different projections, including anterior, lateral and posterior, for IMT measurement. Images of the common carotid artery were recorded for off-line IMT measurement as previously described [16]. Briefly, IMT, which is defined as the distance between the leading edge of the lumen-intima interface and the inner edge of the media-adventitia interface of the far wall, was measured 1 cm proximal to the bulb in the three projections at the end of the systole of the cardiac cycle. The average of the three measurements of IMT (mean IMT) for each side was calculated and was used for statistical analyses along with the maximal IMT (max IMT). The absolute IMT change was calculated using the formula: (IMT follow-up)—(IMT baseline).

### Endothelial function (FMD)

Arterial function was evaluated at the brachial artery of the nondominant arm by FMD technique. The artery was imaged ≈10 cm above the elbow. The gain setting and transducer position were adjusted until a clear image showing that the near and far intima–lumen interface was obtained. After that, the skin was marked, and the transducer was clamped in a probe holder throughout the study. To evaluate endothelial function, a pneumatic cuff was placed around the forearm and inflated to 250 mmHg for 5 min. After deflating the cuff (reactive hyperemia), brachial artery imaging was recorded for 3 min. Images of the artery at baseline, and 50 s, and 2 and 3 min after cuff deflation were captured and analyzed for off-line internal brachial artery diameter (ID) measurement. ID was defined as the distance between intima–lumen interface of the near wall and lumen–intima interface of the far wall.

Brachial artery ID was measured at the end of the diastole using dedicated software (Autodesk Design Review). The software allows careful measurements of distances between selected points. An expert single-blind investigator performed the analysis. Images were captured from the recording of brachial artery at baseline, 50 s, and 2 and 3 min after cuff deflation were displayed on the computer screen. For each image, ID was measured at three different locations of the vessel wall using a caliper placed manually by the operator. The average of three measurements was calculated. The caliper automatically revealed the distance from the intima-lumen interface of the near wall and the lumen-intima interface of the far wall. The caliper distance was calibrated based on the known distance scale displayed on the echo-Doppler screen. FMD was expressed as percentage change of arterial diameter from baseline to post reactive hyperemia and calculated using the following formula: {[postdeflation (50 s, 2, 3 min) ID-baseline ID/baseline ID]x100}. The CV of FMD measurement was ≈6%[15]. Peak dilation was defined as the maximal dilation calculated among three observations [17–18].

### Statistical analysis

Data were analyzed using SPSS 23.0 for Macintosh (SPSS, Inc., Chicago, IL). All variables measured at *Baseline* and *Follow-up Visit* were compared. Triglycerides and FMD were not normally distributed; therefore, they were transformed (log transformation, and 2-step rank transformation) before applying parametric tests. Subjects were divided in two groups: those presenting ≥1 episodes during their observation time were defined as the ‘*H-group*’ (Hypoglycemia group), and those without hypoglycemia were defined as ‘*C-group*’ (Control group). The *t*-*test* for unpaired data was used to compare variables between *C-group* and *H-group* subjects. The *t*-*test* for paired data was used to compare variables measured at *Baseline* and *Follow-up Visit* in each of the two groups. The *chi-square* test was used to compare percentage between two groups. The *General Linear Model* was used to compare IMT measured at *Baseline* and *Follow-up Visit* in the two groups after adjustment for age, sex, and baseline IMT. Stepwise linear regression analysis was performed to evaluate variables independently associated with change in mean IMT. The absolute mean IMT difference (baseline mean IMT–follow-up mean IMT) was the dependent variable, and age, sex, disease duration, number of hypoglycemic events, absolute FPG difference, absolute HbA1c difference, presence or absence of hypoglycemia were independent variables. The sample size was calculated using the variance of FMD technique, which is the method with the highest variability in terms of reproducibility. Therefore, to test the reproducibility of the FMD, 5 subjects were studied five times on different occasions, and the standard deviation (SD) was calculated for each subject. The overall mean SD was 5.2%. Based on the hypothesis of at least a 3% difference in FMD between the two groups after 1 year of observation as well as alpha = 0.05 and beta = 0.10, the number of subjects that needed to be enrolled in the study was estimated to be 60 (30 for each group). A larger number of patients were enrolled to account for an uneven distribution between the two groups and possible drop-outs.

## Results

Seventy-six subjects were enrolled and completed the study. In total, 66% were men, and the overall age range and mean±SD were 46–75 and 60±7 years, respectively. Only 5 subjects were current smokers: 3 in the *C-group* and 2 in the *H-group*.

Based on the presence or absence of hypoglycemic events, 31 (41%) subjects were included in the *H-group*, and 45 (59%) were included in the *C-group*. Mean±SD and median value of hypoglycemic events were 5.8±5.2 and 4.0, respectively. The rate of events calculated as number/person/year was 0.47. Most of events were documented as symptomatic with blood glucose ≤70 mg/dL; 7 subjects documented at least one symptomatic event with blood glucose ≤60 mg/dL. No severe hypoglycemia or asymptomatic hypoglycemia occurred during the study.

Table 1 shows variables measured at *Baseline* and *Follow-up Visit* in patients according to presence or absence of hypoglycemia. Some differences, albeit not statistically significant, were detected between the two groups when comparing data at *Baseline* and *Follow-up Visit*. The prevalence of men was significantly higher than women in the *C-group*. In the *H-group*, fasting plasma glucose and HbA1c significantly decreased from *Baseline* to the *Follow-up Visit*. In the *C-group*, HbA1c and LDL cholesterol significantly decreased from *Baseline* to the *Follow-up Visit*. No statistically significant difference was found for other variables. Antihyperglycemic treatment was comparable between groups. In general, at the follow-up visit, most of the subjects had potentiated the baseline therapy. The number of subjects taking metformin alone slight decreased, while the number of those taking metformin plus incretin-based therapy slightly increased. Sixty-six % of subjects enrolled in the study were taking antihypertensive drugs, and 63% were taking lipid lowering drugs.

**Table 1:** Clinical and biochemical parameters in *C-group* and *H-group* at *Baseline* and *Follow-up Visit*.

	*C-group*		*H-group*	
*Variable*	*Baseline*	*Follow-up*	*Baseline*	*Follow-up*
Number	45	45	31	31
Age (years)	60±6	—	60±8	—
Sex (% male)	75#	—	51	—
Disease duration (years)	9.4±5.8	—	10.8±6.5	—
BMI (kg/m2)	29.9±3.9	29.7±4.1	31.4±4.8	31.2±4.6
SBP (mmHg)	132±16	133±20	133±17	133±18
DBP (mmHg)	82±7	81±9	81±12	81±10
FPG (mmol/L)	8.8±2.9	8.2±0.5	8.1±1.4	7.5±1.5*
HbA1c (%)	7.3±1.2	6.9±0.8*	7.3±0.6	6.7±0.6^
Total cholesterol (mmol/L)	4.4±0.9	4.1±0.7	4.3±0.9	4.2±0.8
HDL cholesterol (mg/dL)	1.2±0.3	1.2±0.3	1.2±0.3	1.3±0.2
LDL cholesterol (mg/dL)	2.7±1.3	2.1±0.6*	2.3±0.8	2.4±0.6
Triglycerides (mg/dL)	1.4±0.8	1.6±1.3	1.6±1.2	1.6±0.9
Metformin (nr)	16	12	11	9
Metformin+Incretin-based therapy (nr)	14	17	11	13
Metformin +Glinid (nr)	6	6	4	4
Basal insulin+OAD (nr)	7	8	4	4
Glinid+Incretin-based therapy (nr)	1	1	0	0
Basal+bolus insulin (nr)	1	1	1	1

OAD oral hypoglycemic agents;

*p<0.03 vs. *Baseline*;

^p<0.0001 vs. *Baseline*;

^#^ p<0.02 vs. *H-group*

Based on the results of the preliminary scan of carotid arteries, 27 (60%) subjects in the *C-group* and 18 (58%) in the *H-group* had a plaque without any stenosis detectable by the Doppler spectrum. Right and left mean and maximum IMT of the common carotid artery measured at *Baseline* and 1-year *Follow-up* were grouped, and results are displayed in Table 2. Baseline mean and maximum IMT were significantly higher in the *C-group* than in the *H-group*. Mean and maximum IMT significantly decreased in the *H-group* from *Baseline* to the *Follow-up Visit* adjusted by age, sex, and baseline IMT. No statistically significant difference was detected in the *C-group*.

**Table 2:** Common carotid artery intima plus media thickness (IMT) in *C-group* and *H-group* patients at baseline and follow-up visit after adjustment for age, sex, and baseline IMT.

	*C-group*		*H-group*	
*Variable*	*Baseline*	*Follow-up*	*Baseline*	*Follow-up*
Number	90	90	62	62
CCA mean IMT (μ)	789±17	809±14	836±135	756±13*#
CCA max IMT (μ)	877±21	869±15	918±131	825±14^#

Data are expressed as the mean±SE;

*p<0.01 vs. baseline;

^p<0.04 vs. baseline;

^#^p<0.02 vs. *C-group*

**Fig 1:**
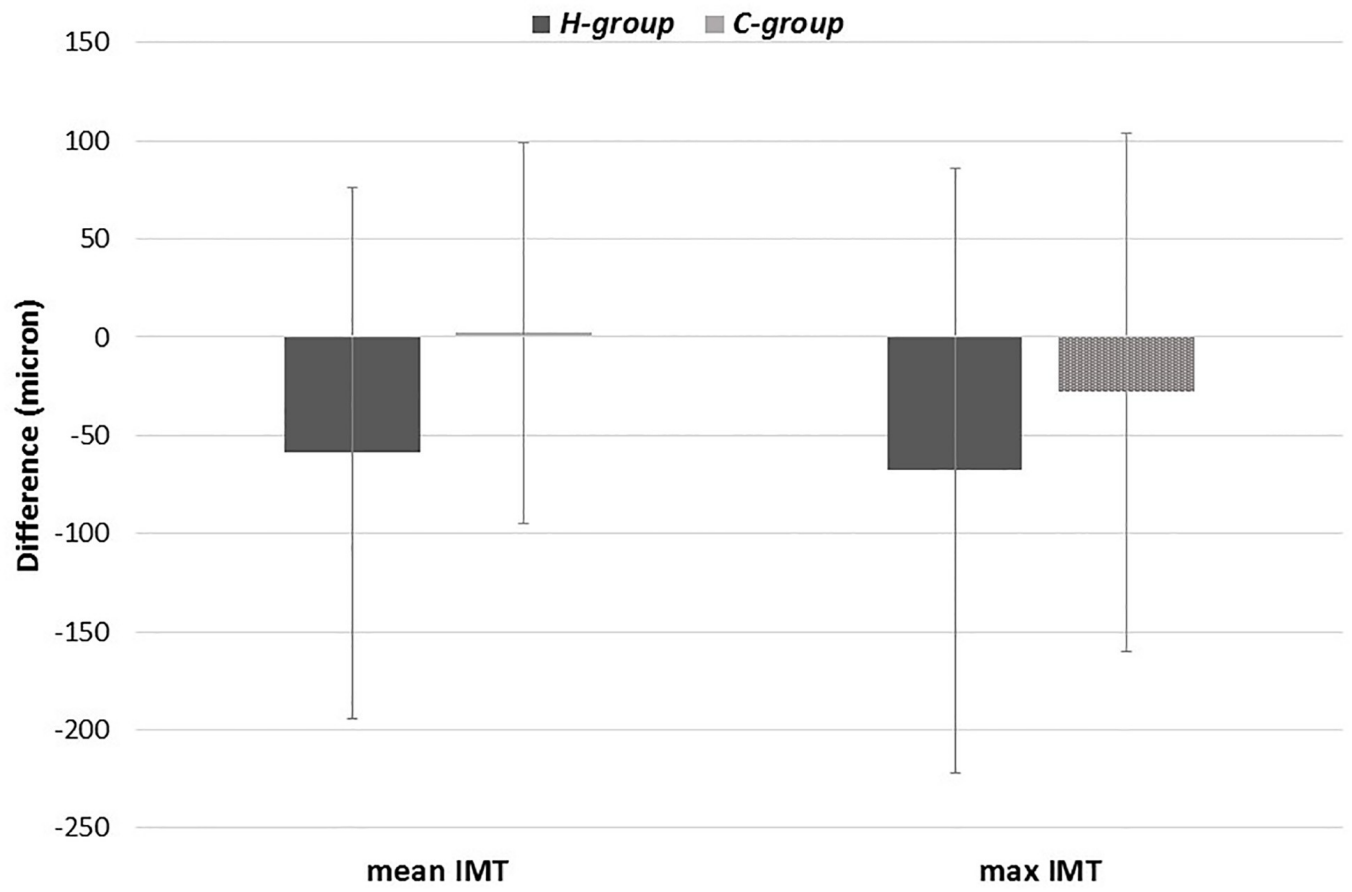
Percent change of mean and maximum IMT in the *C-group* and *H-group*. Right and left arteries have been grouped.

We then calculated the unadjusted mean and maximum IMT absolute difference between *Follow-up* and *Baseline* in both groups, and the results are reported in Fig 1. In detail, in the *H-group*, the mean IMT change was -59±135 μ, and the max IMT change was -68±154 μ. In the *C-group*, the mean IMT change was +2.1±97 μ, and the max IMT change was -28±132 μ.

Hypoglycemia (*H-group*) was significantly associated with 1-year mean IMT change after adjustment for age, sex, disease duration, absolute FPG difference, and absolute HbA1c difference. The number of hypoglycemic events was not independently associated with IMT change. Beta coefficients were calculated by both grouping the right and left sides and separately for each side. Beta coefficients were -85 μ for all arteries (p<0.0001), —75 μ for the right side (p = 0.001), and -88 μ for the left side (p = 0.001).

Baseline brachial artery diameter and FMD at 1 min after ischemia and peak FMD are shown in Table 3. No statistically significant difference was detected between *Baseline* and *Follow-up Visit* in the *H-group* and *C-group*. Furthermore, values at *Baseline* and *Follow-up Visit* were not significantly different between the two groups.

**Table 3:** Baseline brachial artery diameter, FMD 1 min, and Peak FMD in *C-group* and *H-group* at *Baseline* and *Follow-up Visit*.

	*C-group*		*H-group*	
*Variable*	*Baseline*	*Follow-up*	*Baseline*	*Follow-up*
Baseline diameter (mm)	3.7±0.5	3.7±0.4	3.8±0.7	3.7±0.7
FMD 1 min (%)	4.8±8.6	5.6±6.8	4.1±5.6	6.1±6.1
Peak FMD (%)	5.7±7.1	7.7±7.1	6.1±6.2	8.1±6.6

**Fig 2:**
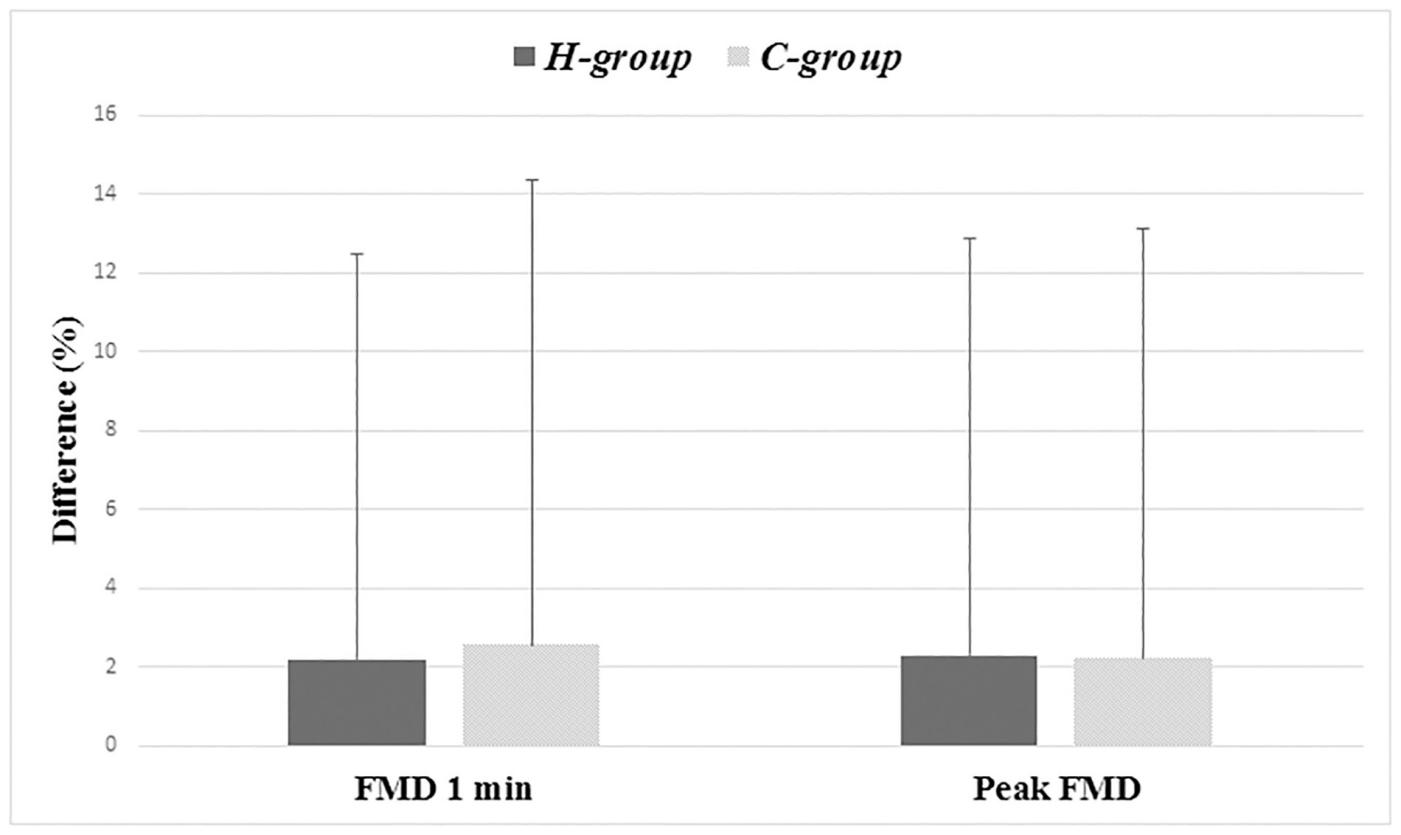
Absolute difference of FMD 1 min and peak FMD after 1 year of observation.

Fig 2 shows the absolute difference of FMD 1 min and peak FMD after 1 year of observation.

## Discussion

The main results of the present study suggest that hypoglycemic events do not cause deterioration of endothelial function, and they can even lead to a reduction of the intima media thickness of the common carotid artery in 60-year-old patients with T2DM and without longstanding uncontrolled hyperglycemia.

Our data appear to be at odds with those of previous studies. Gimenez et al. reported that repeated episodes of hypoglycemia could be considered an aggravating factor for preclinical atherosclerosis in type 1 Diabetes patients [13]. However, the patients enrolled in the Gimenez study differ from the patients in the present study. The Gimenez study included 45 young subjects with T1DM who were in optimal metabolic compensation (HbA1c between 6.6 and 6.7%), were of normal weight, were nonsmokers, and had normal values of blood pressure and of blood lipids. In this situation, patients with hypoglycemia most likely have a high glucose fluctuation. The role of this feature remains controversial but could emerge as a risk factor in otherwise completely healthy subjects without any cardiovascular risk factor[19]. In our study, patients have T2DM, are older, and exhibit the cardiovascular risk pattern typically observed in diabetic subjects at approximately 60 years of age. Although their metabolic compensation is good, the HbA1c values are significantly higher than those reported in the Gimenez study. In this situation, hypoglycemic episodes could simply reflect a better metabolic compensation, which is also partially suggested by the more marked reduction of HbA1c in the group with hypoglycemia compared with the control group.

Peña et al. evaluated endothelial function in subjects with T1DM who underwent continuous glucose monitoring [20]. The enrolled patients were very young (mean age 14 years) without cardiovascular risk factors. The authors found that hypoglycemia but not glycemic variability correlates with reduced endothelial function. Again, the enrolled population is completely different from the one we studied, and this difference could account for the different results.

Taken together, these data suggest that hypoglycemia and glycemic variability may play different roles in patients with type 1 diabetes compared with those with type 2 diabetes.

However, in the case of patients with type 2 diabetes, the selection of subjects can influence the outcome, and it is not surprising that studies comparable in terms of aim achieve different results. Indeed, the regression or progression of diabetic complications is strongly influenced by a patient’s age, disease duration, glycemic control and variability, and comorbidities.

In a post hoc analysis of subjects recruited in the SPIKE trial, a clinical trial designed to investigate the efficacy of sitagliptin versus conventional therapy in controlling the progression of carotid IMT in a Japanese population of type 2 diabetes subjects, the authors analyzed the relationship between hypoglycemic events and change in carotid IMT [21]. They found a significant increase in IMT in subjects with hypoglycemia compared to those without after adjustment for several variables regardless of the treatment. In detail, IMT increased in hypoglycemic subjects by +12 μ in those administered sitagliptin and by +42 μ in those administered conventional therapy. The difference was not statistically significant. Conversely, subjects without hypoglycemia exhibited reduced carotid IMT, and the difference between those taking sitagliptin and conventional therapy was statistically significant (respectively, -57 μ and +12 μ, p = 0.003). In our study, we found a mean IMT regression of approximately -70 μ in subjects with hypoglycemia after 1 year of treatment. The percentage reduction was approximately 6%, a value comparable to that reported in studies investigating the effect of statins on carotid IMT[22]. In addition to the different ethnic origins, subjects in the SPIKE trial were older and had longer disease duration and higher baseline HbA1c compared with subjects enrolled in our study. They suffered from severe renal disease, had less endogenous insulin and were frequent users of sulfonylureas. The populations the authors have investigated are characterized by an advanced stage of disease and higher cardiovascular risk. We believe that these different characteristics may have influenced the different outcome of our study compared with the SPIKE Trial.

The findings of our study have also demonstrated a lack of worsening of endothelial function in the *H-group* compared with the *C-group* after 1 year of treatment. Endothelial function has been not extensively investigated in subjects with hypoglycemia. The acute effect of moderate hypoglycemia on endothelial function has been investigated in healthy non-diabetic individuals [23]. The authors reported blunted dilation during acute hypoglycemia and acute hyperglycemia. They also found a significant increase in pro-inflammatory and pro-atherothrombotic cytokines in both conditions. Both results make sense and suggest how relevant and unsafe might be an acute hypo- and hyperevent. In the present paper, we demonstrated that hypoglycemic events did not negatively influence the vascular function.

The prevalence and incidence of hypoglycemic events observed in the present study is comparable to that reported in epidemiological studies and meta-analyses [24,25], and in our opinion, this finding particularly strengthens the findings of the present study.

The paper has some limitations. In particular, we have likely underestimated the number of hypoglycemic events. Indeed, it is possible that not all events and the unawareness episodes have been documented. This is a limit of all studies that include hypoglycemia as outcome and is only avoidable with the use of continuous glucose monitoring. The results should be confirmed in a larger population.

In conclusion, the results of the present study suggest that despite a reasonable concern for hypoglycemia, each strategy aimed to obtain a good metabolic control in nonfragile subjects with type 2 diabetes should be pursued. Our data demonstrated for the first time that optimal glycemic control, even if associated with increased rate of hypoglycemic events, does not affect the progression of preclinical atherosclerosis in subjects without previous severe hypoglycemia and with known micro- and macrovascular complications.

Clinical characteristics, type of diabetes, presence of complications, disease duration and previous metabolic control might suggest the metabolic target and the intensity of treatment after evaluating the risk of hypoglycemia in terms of morbidity and mortality.

## References

[1] Intensive diabetes treatment and cardiovascular outcomes in type 1 diabetes: The DCCT/EDIC study 30-year follow-up. Diabetes Control and Complication Trial DDCT/Epidemiology of Diabetes Interventions and Complications (EDIC) Study Research Group. Diabetes Care. 2016;39:686–693. 10.2337/dc15-1990 26861924 PMC4839174

[2] Holman RR, Paul SK, Bethel MA, Matthews DR, Neil HA. 10-year follow-up of intensive glucose control in type 2 diabetes. N Engl J Med. 2008;359:1577–1589. 10.1056/NEJMoa0806470 18784090

[3] Gerstein HC, Miller ME, Byinngton RP, Goff DC, Bigger JT, Buse JB, et al Effect of intensive glucose lowering in type 2 diabetes. The Action to Control Cardiovascular Risk in Diabetes Study Group. N Engl J Med. 2008;358:2545–2559. 10.1056/NEJMoa0802743 18539917 PMC4551392

[4] Duckworth W, Abraira C, Moritz T, Reda D, Emanuele N, Reaven PD, et al for the VADT Investigators. Glucose control and vascular complications in veterans with type 2 diabetes. N Engl J Med. 2009;360:129–139. 10.1056/NEJMoa0808431 19092145

[5] Patel A, MacMahon S, Chalmers J, Neal B, Billot L, Woodward M, et al for the ADVANCE Collaborative Group. Intensive blood glucose control and vascular outcomes in patients with type 2 diabetes. N Engl J Med. 2008;358:2560–2572. 10.1056/NEJMoa0802987 18539916

[6] Bonds DE, Miller ME, Bergenstal RM, Buse JB, Byington RP, Cutler JA, et al The association between symptomatic, severe hypoglycaemia and mortality in type 2 diabetes: retrospective epidemiological analysis of the ACCORD study. BMJ. 2010;340:b4909 10.1136/bmj.b4909 20061358 PMC2803744

[7] Skyler JS, Bergenstal R, Bonow RO, Buse J, Deedwania P, Gale EA, et al Intensive glycemic control and the prevention of cardiovascular events: implications of the ACCORD, ADVANCE, and VA Diabetes Trials: a position statement of the American Diabetes Association and a scientific statement of the American College of Cardiology Foundation and the American Heart Association. J Am Coll Cardiol. 2009;53:298–304. 10.1016/j.jacc.2008.10.008 19147051

[8] Yang SW, Park KH, Zhou YJ. The impact of hypoglycemia on the cardiovascular system: physiology and pathophysiology. Angiology 2016;67:802–809. 10.1177/0003319715623400 26685181

[9] Zammitt NN, Frier BM. Hypoglycemia in type 2 diabetes: pathophysiology, frequency, and effects of different treatment modalities. Diabetes Care. 2005;28:2948–2961 16306561 10.2337/diacare.28.12.2948

[10] McCrimmon RJ, Sherwin RS. Hypoglycemia in type 1 diabetes. Diabetes Care. 2010;59:2333–2339. 10.2337/db10-0103 PMC3279554 20876723

[11] Desouza CV, Bolli GB, Fonseca V. Hypoglycemia, diabetes, and cardiovascular events. Diabetes Care. 2010;33:1389–1394. 10.2337/dc09-2082 20508232 PMC2875462

[12] Mita T, Katakami N, Shiraiwa T, Yoshii H, Kuribayashi N, Osonoi T, et al Relationship between frequency of hypoglycemic episodes and change in carotid atherosclerosis in insulin-treated patients with type e diabetes mellitus. Scientific Report 2017;7:39965 10.1038/srep39965 28067320 PMC5220284

[13] Gimenez M. Gilabert R, Monteagudo J, Alonso A, Casamitjana R, Parè C, et al Repeated episodes of hypoglycemia as a potential aggravating factor for preclinical atherosclerosis in subjects with type 1 diabetes. Diabetes Care 2011;34: 198–203. 10.2337/dc10-1371 20929996 PMC3005490

[14] Touboul PJ, Hennerici MG, Meairs S, Adams H, Amarenco P, Bornstein N, et al. Mannheim carotid intima-media thickness and plaque consensus (2004-2006-2011). Anupdate on behalf of the advisory board of the 3rd, 4th and 5th watching the risksymposia, at the 13th, 15th and 20th European Stroke Conferences, Mannheim, Germany, 2004, Brussels, Belgium, 2006, and Hamburg, Germany, 2011. Cerebrovasc Dis. 2012;34:290–296. 10.1159/000343145 PMC3760791 23128470

[15] Gokaldas R, Singh M, Lal S. Carotid stenosis: from diagnosis to management, where do we stand? Curr Atheroscler. 2015; 10.1007/s11883-014-0480-7 25609266

[16] Irace C, Carallo C, De Franceschi MS, Scicchitano F, Milano M, Tripolino C, et al Human common carotid wall shear stress as a function of age and gender: a 12-year follow-up study. Age (Dordr). 2012;34:1553–1562. 21989971 10.1007/s11357-011-9318-1 PMC3528365

[17] Irace C, Tschakovsky ME, Carallo C, Cortese C, Gnasso A. Endothelial dysfunction or dysfunctions? Identification of three different FMD responses in males with type 2 diabetes. Atherosclerosis.2008;200:439–445. 10.1016/j.atherosclerosis.2007.12.036 18262189

[18] Irace C, De Rosa S, Tripolino C, Ambrosio G, Covello C, Abramo E, et al Delayed flow-mediatedvasodilation and criticalcoronarystenosis. J Investig Med. 2018: 66:905–911 10.1136/jim-2017-000644 29550752

[19] Kilpatrick ES, Rigby AS, Atkin SL. Mean blood glucose compared with HbA1c in the prediction of cardiovascular disease in patients with type 1 diabetes. Diabetologia. 2008; 51:365–371. 10.1007/s00125-007-0883-x 18040661

[20] Peña AS, Couper JJ, Harrington J, Gent R, Fairchild J, Tham E, et al Hypoglycemia, but not glucose variability, relates to vascular function in children with type 1 diabetes. Diabetes Technol Ther. 2012;14:457–462. 10.1089/dia.2011.0229 22313018 PMC3359626

[21] Mita T, Katakami N, Shiraiwa T, Yoshii H, Onuma T, Kuribayashi N. Sitagliptin Attenuates the Progression of Carotid Intima-Media Thickening in Insulin-Treated Patients With Type 2 Diabetes: The Sitagliptin Preventive Study of Intima-Media Thickness Evaluation (SPIKE): A Randomized Controlled Trial. Diabetes Care. 2016;39:455–464. 10.2337/dc15-2145 26822324

[22] Paraskevas KI, Hamilton G, Mikhailidis DP. Statins: an essential component in the management of carotid artery disease. J Vasc Surg. 2007;46:373–386. 10.1016/j.jvs.2007.03.035 17664116

[23] Joy NG, Perkins JM, Mikeladze M, Younk L, Tate DB, Davis SN. Comparative effects of acute hypoglycemia and hyperglycemia on pro-atherothrombotic biomarkers and endothelial function in non-diabetic humans. Journal of Diabetes and Its Complications. 2016;30:1275–1281. 10.1016/j.jdiacomp.2016.06.030 27445005 PMC4987190

[24] Chloe LE, Dunkley AJ, Bodicoat DH, Rose TC, Gray LJ, Davies MJ. Prevalence and Incidence of Hypoglycaemia in 532,542 People with Type 2 Diabetes on Oral Therapies and Insulin: A Systematic Review and Meta-Analysis of Population Based Studies. PlosONE. 2015; 10.1371/journal.pone.0126427 26061690 PMC4465495

[25] Elliott E, Fiedler C, Ditchfield A, Stissing T. Hypoglycemia event rates: a comparison between real word evidence data and randomized controlled trial populations in insulin treated diabetes. Diabetes Ther. 2016;7:45–60. 10.1007/s13300-016-0157-z 26886441 PMC4801820

